# Efficacy of diaphragmatic breathing exercise on respiratory, cognitive, and motor function outcomes in patients with stroke: a systematic review and meta-analysis

**DOI:** 10.3389/fneur.2023.1233408

**Published:** 2024-01-12

**Authors:** Auwal Abdullahi, Thomson WL Wong, Shamay SM Ng

**Affiliations:** Department of Rehabilitation Sciences, The Hong Kong Polytechnic University, Hong Kong, China

**Keywords:** stroke, breathing exercises, vital capacity, forced expiratory volume, maximal respiratory pressures, activities of daily living, quality of life

## Abstract

**Background:**

Stroke disrupts the functions carried out by the brain such as the control of movement, sensation, and cognition. Disruption of movement control results in hemiparesis that affects the function of the diaphragm. Impaired function of the diaphragm can in turn affect many outcomes such as respiratory, cognitive, and motor function. The aim of this study is to carry out a systematic review and meta-analysis to determine the efficacy of diaphragmatic breathing exercise on respiratory, cognitive, and motor outcomes after stroke.

**Method:**

The study was registered in PROSPERO (CRD42023422293). PubMED, Embase, Web of Science (WoS), PEDro, Scopus, and CENTRAL databases were searched until September 2023. Only randomized controlled trials comparing diaphragmatic breathing exercise with a control were included. Information on the study authors, time since stroke, mean age, height, weight, sex, and the protocols of the experimental and control interventions including intensity, mean scores on the outcomes such as respiratory, cognitive, and motor functions were extracted. Cochrane risks of bias assessment tool and PEDro scale were used to assess the risks of bias and methodological quality of the studies. Narrative synthesis and meta-analysis were used to summarize the results, which were then presented in tables, risk-of-bias graph, and forest plots. The meta-analysis was carried out on respiratory function [forced vital capacity (FVC), forced expiratory volume in 1 s (FEV1), FEV1/FVC, peak expiratory flow (PEF)] and motor function (trunk impairment, and internal and external oblique muscles activity).

**Results:**

Six studies consisting of 151 participants were included. The results of the meta-analyses showed that diaphragmatic breathing exercise is only superior to the control at improving respiratory function, FVC (MD = 0.90, 95% CI = 0.76 to 1.04, *P* < 0.00001), FEV1 (MD = 0.32, 95% CI = 0.11 to 0.52, *P* = 0.002), and PEF (MD = 1.48, 95% CI = 1.15 to 1.81, *P* < 0.00001).

**Conclusion:**

There is limited evidence suggesting that diaphragmatic breathing exercise may help enhance respiratory function, which may help enhance recovery of function post stroke.

**Systematic Review Registration:**

PROSPERO, identifier CRD42023422293.

## Introduction

Stroke disrupts movement, sensation, and cognition functions that are normally carried out by the brain (1–3). One of the movement functions controlled by the brain is that of the trunk muscles, which gets impaired after stroke as a result of hemiparesis (4–7). The trunk muscles, especially the diaphragm, contribute to respiration by moving the chest up and down (8). However, following a stroke, it was noted that approximately 52% of the patients developed dysfunction of the diaphragm, such as reduced diaphragmatic excursion, that eventually resulted in respiratory compromise (4, 9). Consequently, compared with the healthy control, stroke survivors have lower values of forced vital capacity (FVC), forced expiratory volume in 1 s (FEV1), peak expiratory flow (PEF), and diaphragmatic excursion (10).

Diaphragmatic excursion, movement of the diaphragm during breathing, directly and indirectly influences the parasympathetic and sympathetic nervous systems, consequently impacting the activities of the motor nerves (11, 12). This is because the diaphragm is supplied by the phrenic nerve, which is closely connected with the vagus nerve, the longest nerve in the body that controls many functions in the body (12). Thus, diaphragmatic breathing exercise, which is a type of breathing exercise that involves slow, smooth, and deep inspiration through the nose, accompanied by the displacement of the abdomen together with the diaphragm can be used to improve respiratory and other functions after stroke (13–16). It is usually carried out in supine position (sometimes with trunk inclination of approximately 30 degrees to help enhance the action of the diaphragm) with one hand placed on the chest to allow for only a minimal movement of the chest and the other hand on the belly (17, 18).

The main effects of diaphragmatic breathing exercise include increased tidal volume and oxygen saturation, reduction in breathing frequency, and improvement in ventilation and hematosis (19, 20). Normalization of breathing results in delivery of adequate oxygen to body tissues for many metabolic activities (21, 22). Consequently, it has been used to improve stress, attention, negative affect, FVC, FEV1, PEF, and head posture in both health and disease (23, 24). Thus, diaphragmatic breathing exercise seems to play role in improving respiratory, cognitive, and motor functions in patients with stroke. Improvement in respiratory, cognitive, and motor functions after stroke are related to the patients' ability to carry out activities of daily living (ADL), participate in activities, and to achieve increased quality of life (25–30). The aim of this systematic review and meta-analysis is to determine the evidence from the literature on the efficacy of diaphragmatic breathing exercise on respiratory, cognitive, and motor outcomes after stroke.

## Materials and methods

This study is a systematic review and meta-analysis of the literature on the efficacy of diaphragmatic breathing exercise on outcomes after stroke. The review was conducted following the Preferred Reporting Items for Systematic Reviews and Meta-Analyses (PRISMA) guidelines. For transparency in the process of conducting the review, it was registered in PROSPERO. The registration number is CRD42023422293.

### Criteria for inclusion of studies in the review

The inclusion criteria used in the study is based on participants (patients with stroke who are 18 years or older), intervention (diaphragmatic breathing exercise), comparator (a control intervention such as usual care or any other intervention other than diaphragmatic breathing exercise), and outcomes (respiratory, cognitive, and motor functions). Studies were only included if they are randomized controlled trials (RCTs). However, studies that were published in languages other than English were excluded from the review.

### Searching the literature

The search was carried out in PubMED, Embase, Web of Science (WoS), PEDro, Scopus, and CENTRAL from the inception of each database until September 2023. The search terms used are stroke, breathing exercises forced vital capacity, maximal expiratory pressure, maximal cognitive function, and motor activity. Each search was adapted to the specific requirement of the databases. The search strategy used is provided in the Appendix.

In addition, a manual search of the reference lists of the included studies and relevant systematic reviews was carried out. The search was performed by AA but was independently confirmed by TWLW.

### Study selection

Eligible studies were independently selected using Endnote software by AA and TWLW. Initially, the selection was carried out based on titles and abstracts of the studies. In that case, studies that were deemed ineligible were immediately excluded. Subsequently, the full texts of the remaining studies were read to determine their eligibility. Following this, AA and TWLW held a meeting to agree on their independent selections of the studies. However, when they were unable to agree on the selection of a particular study, SSMN was contacted to help resolve the disagreement.

### Data extraction

Information on the study authors, time since stroke, mean age, height, weight, sex, and the protocols of the experimental and control interventions including the intensity (how many minutes or hours per day, how many times a week, and for how many weeks the breathing exercise was carried out), mean scores on the outcomes of interest such as respiratory function [FVC, FEV1, FEV1/FVC, PEF, oxygen saturation (SPO_2_), maximal inspiratory pressure (MIP), and maximal expiratory pressure (MEP)], cognitive function, and motor function (muscle activation or activity and trunk impairment), chest circumference, and abdominal muscle thickness and balance were extracted by AA. However, TWLW and SSMN independently verified the extracted data for quality assurance. The extracted data were stored in a Microsoft Excel file.

### Risks of bias and methodological quality assessments

The Cochrane Risk of Bias Assessment tool was used to assess the risks of bias of the included studies. It is a valid and reliable instrument that assesses selection, performance, detection, attrition, and reporting biases in addition to any other action deemed as a bias in the conduct of a study (31).

For the methodological quality assessment, a tool known as PEDro scale that consists of 11 items was used (32). The first item of the tool assesses internal validity, whereas the remaining 10 items assess external validity, which are rated on a two-point scale, 0 (when the answer to an item is no) and 1 (when the answer to an item is yes) (32). The total scores obtained from the scale can be designated as low, moderate, or high quality when it is between 0 and 3, 4 and 5, or 6 and 10, respectively (33–35).

Both the assessments of the risks of bias and methodological quality were carried out by AA and TWLW independently. Following that, they met to agree on their assessments. However, SSMN was involved when they could not agree on a particular assessment.

### Synthesis of the extracted data

The extracted data were synthesized using both qualitative and quantitative syntheses. The qualitative synthesis was used to provide a summary of the characteristics of the participants in the included studies. The quantitative synthesis was carried using both fixed effect model and random effect model meta-analyses to pool together the means and standard deviations of the scores on the outcomes of interest and the study sample sizes in the included studies.

Initially, fixed effect model meta-analysis was used to determine the effect size. The fixed effect model meta-analysis is used based on the assumption that all effect estimates are estimating the same intervention effect (36). In a subsequent step, a sensitivity analysis was carried out using a random-effect model meta-analysis. The random-effect model meta-analysis is used based on the assumption that different studies are estimating different but related intervention effects (36, 37). However, conducting further sensitivity analyses in terms of, for example, time since stroke, the type of outcomes, the interventions used, and other characteristics of the participants in the included studies was not possible due to lack of adequate information. Percentage variation due to heterogeneity between the included studies was determined using *I*^2^ statistics. Consequently, *I*^2^ statistics values between 50 and 90% at *P* < 0.05 were regarded to indicate the presence of significant heterogeneity between the included studies.

The meta-analysis was carried out on the respiratory function (forced vital capacity (FVC), forced expiratory volume in 1 s (FEV1), and FEV1/FVC), and motor function (trunk impairment, and internal and external oblique muscle activity). All the meta-analyses were carried out using RevMan version 5.3 software (38). In addition, we did not require further information, and as such, we did not contact the authors of the included studies.

## Evidence interpretation

The Grading of Recommendations, Assessment, Development, and Evaluation instrument (GRADE) was used to interpret the evidence (39). It is an instrument that consists of five domains: risks of bias, imprecision, inconsistency, indirectness, and publication bias. The evidence is summarized in a table.

## Results

### Selection of the included studies

**Figure 1:**
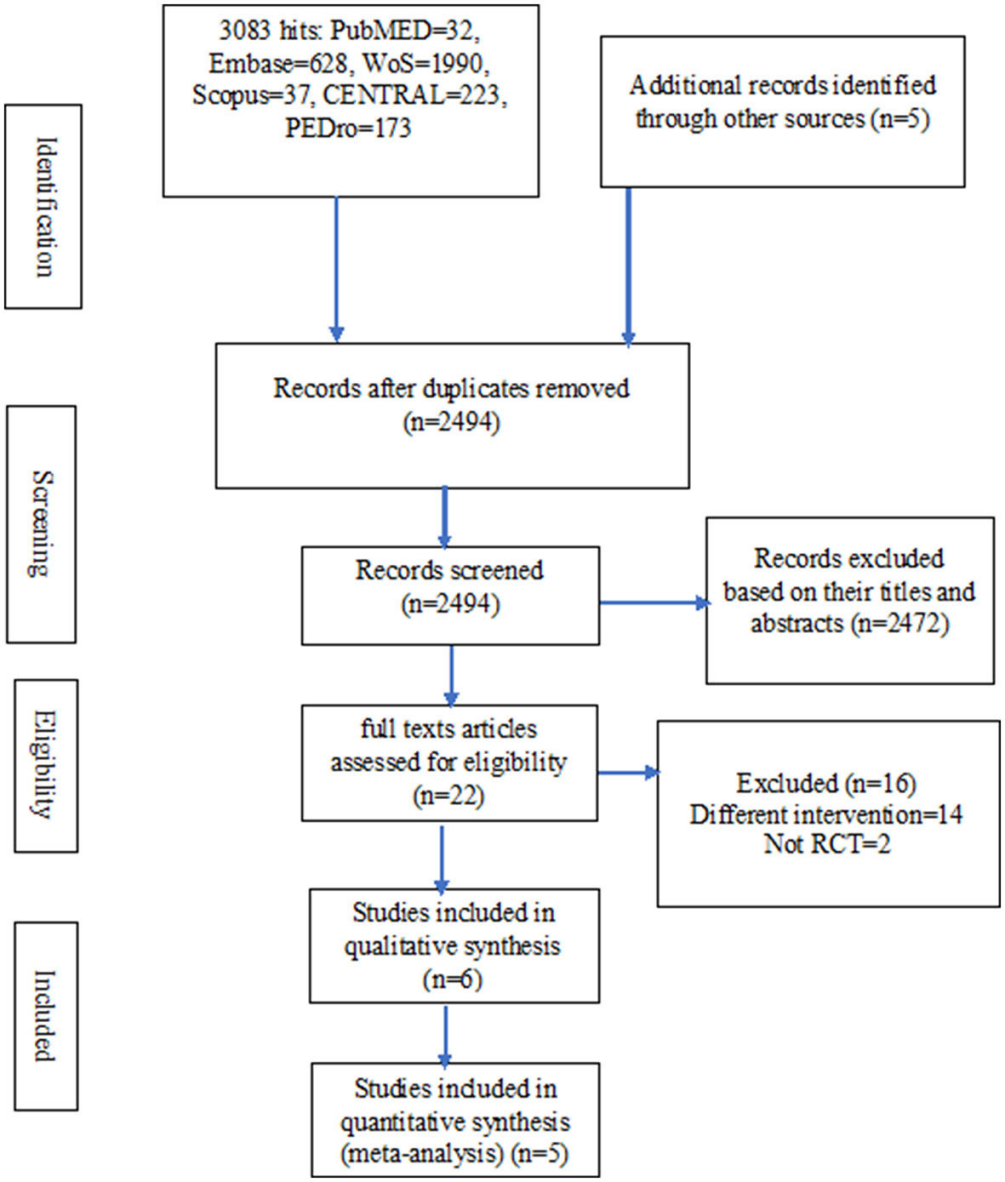
The study flowchart.

In total, there were 3,088 hits from the literature. Out of these hits, only six studies were eligible for inclusion in the review (40–45). However, two other potential eligible studies were excluded for being a non-randomized trial and having multiple interventions including two different breathing exercises in the experimental group, respectively (16, 46). Details of the literature search and the selection of the studies are presented in Figure 1.

### Characteristics of the included studies

In the included studies, there were 151 patients with stroke, out of which 64 were women. The participants have a mean age ranging from 50.10±3.213 to 73.92±7.93 years and mean time since stroke range between 90.33 ± 69.63 days and 20.33± 8.52 months.

Both participants with ischaemic and hemorrahagic types of stroke were included in the studies. However, the information was provided by only three studies, wherein 55 and 31 of the participants had ischemic and hemorrahgic strokes, respectviely (40, 43, 45). Similarly, only five studies provided information on the side affected, 68 on the right side and 49 on the left side (40, 41, 43–45).

Moreover, only five studies provided information on the participants' height and weight, which we used to calculate their mean BMI (40–42, 44, 45). However, in one of the studies, the BMI value is 25.29kg/m^2^ for the experimental group (41), which indicates that they have grade I obesity (47).

For the inclusion criteria, diagnosis of stroke was achieved using computed tomography (CT) and/or magnetic resonance imaging (MRI) in only two studies (41, 43). However, in all the studies, only participants with no significant cognitive impairment were included (40–45). In addition, only four studies provided information on the severity of the disability the included participants had, which was moderate as indicated by the participants' ability to stand independently (40, 41, 43, 45).

For the exclusion criteria, some of the studies excluded participants with musculoskeletal disorders (40–42, 44); previous neurological disorders (40, 43); previous pulmonary disorders (41, 45); cardiac problems (44, 45); pain (40); visual problem (40); and hemineglect (40).

For the differences in the protocols of the included studies, participants performed only diaphragmatic breathing exercise without any additional intervention aside from conventional therapy or usual care in only two studies (40, 43). In the remaining studies, other interventions were added to diaphragmatic breathing exercise (41, 42, 44, 45).

Similarly, the outcomes assessed in the studies include forced vital capacity (FVC) (42, 44, 45); forced expiratory volume in 1 s (FEV1) (42, 44, 45); FEV1/FVC (42, 45); peak expiratory flow (PEF) (42, 44, 45); SPO_2_ (42); motor impairment of the trunk (40, 43); balance (40); cognitive ability (42); muscle activation (41); abdominal muscles thickness (40); and chest circumference (44, 45). Details of the characteristics of the included studies are presented in Table 1.

**Table 1:** Characteristics of the included studies.

**References**	***N***	***Stroke duration***	**Mean age (years)**	**BMI (kg/m^2^)**	**Intervention**	**Outcomes**	**Findings**	**Adverse events**
Kim et al. (40)	Inpatient *N =* 19; experimental (*n =* 9, female = 2); control (*n =* 10, female = 4);	Experimental (16.8 ± 8.6); control (19.3 ± 9.5) months	Experimental (59.1 ± 13.7); control (59.3 ± 10.5)	Experimental (23.95); control (22.87)	Participants in both groups received 60 mins conventional rehabilitation, 3 times a week for 4 weeks. Experimental = diaphragmatic breathing maneuver Control = abdominal drawing-in maneuver In both groups, participants performed the maneuvers and maintained them for 5 seconds with normal breathing, and a 3 second rest afterward. This carried 10 times per session, 3 times a day, 3 times a week for 4 weeks. Between sessions, there was 60 seconds rest.	Abdominal muscle thickness (ultrasound), motor impairment of the trunk (TIS), and balance (BBS).	All outcomes improved post intervention in both groups. However, motor impairment of the trunk improved more significantly in the experimental group.	Not reported
Seo et al. (41)	*N =* 30; experimental (*n =* 15, female = 8); control (*n =* 15, female = 7);	Experimental (9.1 ± 6.8); control (10.3 ± 3.1) months	Experimental (63.6 ± 3.7); control (66.5 ± 8.1)	Experimental (25.29); control (22.72)	Experimental = 15 minutes inspiratory diaphragm breathing and expiratory pursed-lip breathing exercises in addition to conventional physical therapy consisting of joint mobilization, muscular strengthening and extension exercises, 5 times a week for 4 weeks. Control: received conventional physical therapy consisting of joint mobilization, muscular strengthening and extension exercises for 15 minutes, 5 times a week for 4 weeks.	Activation of UT, LD, RA, EAO, and IAO. (EMG).	Significantly improvement in the activation of UT, LD, RA, EAO, and IAO occurred only in the experimental group	Dizziness and fatigue
Kim et al. (44)	*N =* 24; experimental (*n =* 12, female = 2); control (*n =* 12, female = 4)	Experimental (9.33 ± 1.50); control (9.42 ± 2.07) months	Experimental (64.83 ± 13.10); control (65.42 ± 9.71)	Experimental (23.51); control (23.35)	Participants in both groups received the intervention for 30 mins per day, 3 times a week for 4 weeks. Experimental = diaphragmatic breathing exercise + rib cage joint mobilization. Control = diaphragmatic breathing exercise.	FVC, FEV1 and PEF (spirometry); chest circumference (tape measure).	FEV1, PEF and upper chest circumference improved more significantly in the experimental groups.	Not reported
Rasheed et al. (43)	Outpatient *N =* 36; experimental (*n =* 18, female = 9); control (*n =* 18, female = 10);	Experimental (120.56 ± 75.63); control (90.33 ± 69.63) days	Experimental (59.94 ± 9.14); control (55 ± 10.387)	Not reported	Participants in both groups received physical therapy, 5 times a week for 4 weeks. Experimental = In crook lying position, participants inhaled deeply through the nose so that they could see their abdomen expanding and held this position for 5 seconds and then exhaled through mouth, 10 times per session for total of 3 sessions with 1-minute rest between each session. Control = performed abdominal drawing-in maneuver in crook lying position by drawing-in the lower abdomen (below umbilicus) and tilting the pelvis posteriorly and holding the position for 5 seconds, 10 times per session for total of 3 sessions with 1-minute.	Motor impairment of the trunk (TIS)	Trunk motor impairment improved in both groups. However, it improved significantly better in the experimental group compared to the controls.	Not reported
Yoon et al. (45)	*N =* 22; experimental (*n =* 16, female = 6); control (*n =* 16, female = 7)	Experimental (13.31 ± 2.21); control (12.25 ± 2.14) months	Experimental (73.92 ± 7.93); control (71.09 ± 6.80)	Experimental (22.98); control (24.34)	All participants received comprehensive rehabilitation therapy, including central nervous system training and 30-minute walking exercises, five times a week for 4 weeks Experimental = breathing exercise group performed 20 minutes of diaphragmatic breathing in combination with pursed lip breathing exercises, three times a week for 4 weeks Control = performed 20 minutes of non-resistant cycle ergometer exercise three times a week for 4 weeks	FVC, FEV1, FVC/FEV1, PEF (spirometry); chest circumference (MEP-MIP); endurance (6MWT)	Only the experimental group improved FVC, FEV1, PEF and chest circumference post intervention. However, both groups improved endurance post intervention.	Not reported
Mushtaq et al. (42)	*N =* 20; experimental (*n =* 10, female = 2); control (*n =* 10, female = 3)	Experimental (17.33± 7.13); control (20.33± 8.52) months	Experimental (50.10 ± 3.213); control (55.30 ± 5.945)	Experimental (23.03); control (23.20)	Experimental = diaphragmatic breathing exercise with resistance for 30 minutes + 15 minutes receive digital spirometer training Control = 15 minutes receive digital spirometer training Both groups received the interventions 3 times a week for 4 weeks	FVC, FEV1, FVC/FEV1 and PEF (spirometry); SPO_2_ (pulsed oximetry); cognitive ability (K-MMSE)	All outcomes improved post intervention in both groups. However, the improvement in the experimental group is significantly higher than that of the control.	Not reported

FVC, forced vital capacity; FEV1, forced expiratory volume in 1 second; PEF, peak expiratory flow; sEMG, surface electromyography; MIP, maximal inspiratory pressure; MEP, maximal expiratory pressure; FSS, fatigue severity scale; ADL, activities of daily living; FIM, functional independence measure; TIS, trunk impairment scale; BBS, Berg balance scale; K-MMSE, Korean version of minimental state examination; 6MWT, six-minute walk test; UT, upper trapezius; LD, lattismus dorsi; RA, rectus abdominus; EAO, external abdominal oblique; IAO, internal abdominal oblique; EMG, electromyography.

### Methodological quality and risks of bias of the included studies

Among the studies, four have high methodological quality (40, 43–45), while the remaining two have moderate methodological quality (41, 42). Details of the methodological quality of the included studies are presented in Table 2.

**Table 2:** Methodological quality of the included studies.

**Study**	**Eligibility criteria specified**	**Random allocation**	**Concealed allocation**	**Comparable subjects**	**Blind subjects**	**Blind therapists**	**Blind assessors**	**Adequate follow-up**	**Intention to treat analysis**	**Between group comparison**	**Point estimation and variability**	**Total score**
Kim et al. (40)	Yes	1	0	1	0	0	1	1	0	1	1	6/10
Seo et al. (41)	Yes	1	0	1	0	0	0	1	1	0	1	5/10
Kim et al. (44)	Yes	1	0	1	0	0	1	1	1	1	1	7/10
Rasheed et al. (43)	Yes	1	0	1	0	0	1	1	1	1	1	7/10
Yoon et al. (45)	Yes	1	0	1	0	0	0	1	1	1	1	6/10
Mushtaq et al. (42)	Yes	1	0	1	0	0	0	1	1	1	1	5/10

**Figure 2:**
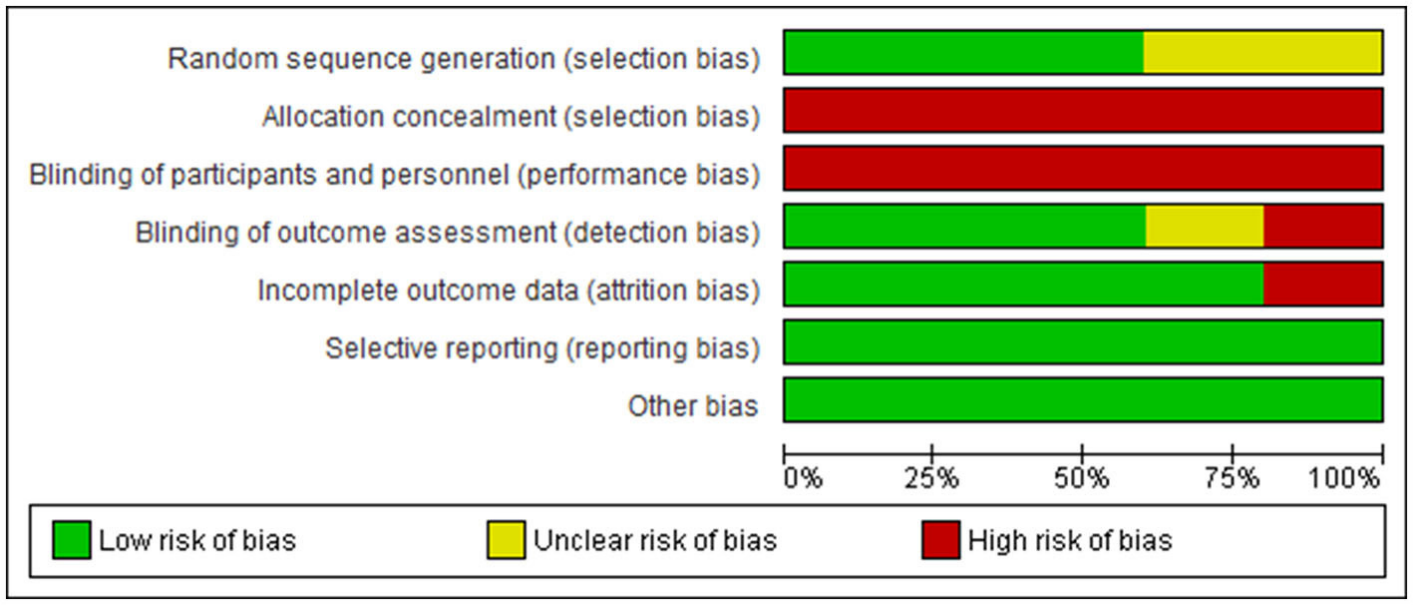
Risk of bias graph.

However, in the studies, there are high risks of bias in allocation concealment (selection bias) (40–45); blinding of participants and personnel (performance bias) (40, 42–45); incomplete outcome data (attrition bias) (40, 42); and blinding of outcome assessment (detection bias) (41, 42, 45). In addition, there is unclear risk of bias in random sequence generation (selection bias) (41, 44) in two of the sudies. See Figure 2 for the risks-of-bias graph.

### Quantitative synthesis

#### Respiratory function

**Figure 3:**
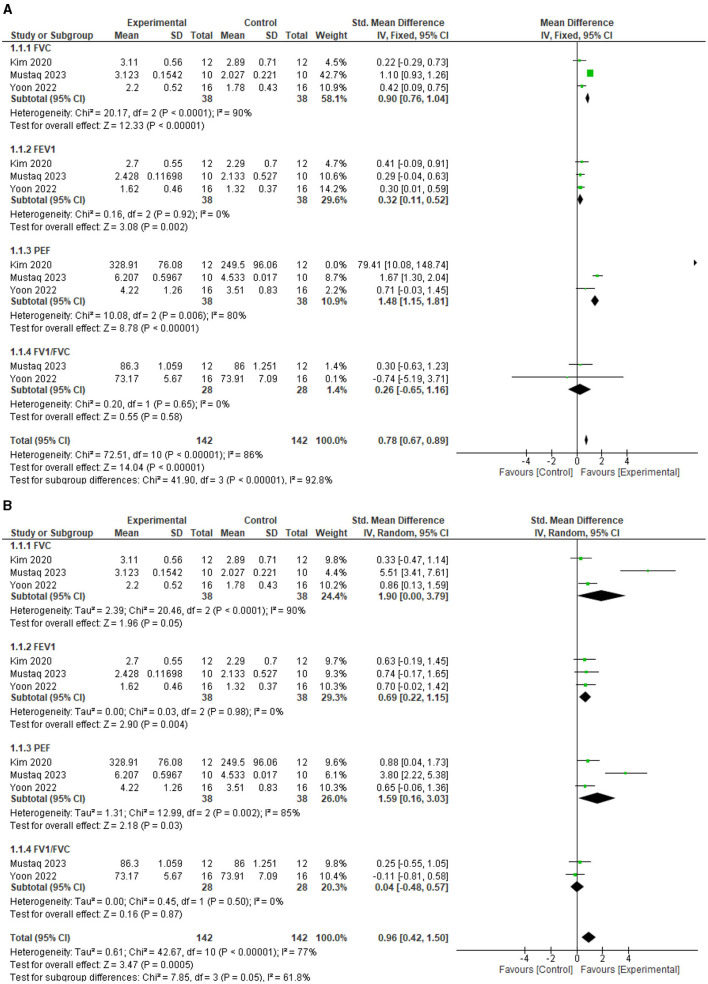
**(A)** Effect of diaphragmatic breathing exercise on respiratory function post-intervention. **(B)** Effect of diaphragmatic breathing exercise on respiratory function post-intervention (sensitivity analysis).

The test for overall effects post intervention showed that respiratory function improved significantly higher in the experimental group compared with the control (MD = 0.78, 95% CI = 0.67 to 0.89, *P* < 0.00001). However, the heterogeneity between the included studies is significant (*I*^2^=86%, *p* < 0.00001). See Figure 3A for the forest plot showing the details of the result. In addition, following sensitivity analysis, the test for overall effects post intervention still showed that respiratory function improved significantly higher in the experimental group compared to the control (SMD = 0.96, 95% CI = 0.42 to 1.50, *P* = 0.0005). See Figure 3B for the details of the result.

For the individual respiratory function parameters, FVC improved significantly better in the experimental group compared with the control (MD = 0.90, 95% CI = 0.76 to 1.04, *P* < 0.00001) post intervention. However, the heterogeneity between the included studies is significant (*I*^2^=90%, *p* < 0.0001). See Figure 3A for the forest plot showing the details of the result.

For FEV1, the experimental group improved significantly better compared to the control (MD = 0.32, 95% CI = 0.11 to 0.52, *P* = 0.002) post intervention. In addition, there is no significant heterogeneity between the included studies (*I*^2^=90%, *p* = 0.92). See Figure 3A for the forest plot showing the details of the result.

For PEF, the experimental group improved significantly better compared to the control (MD = 1.48, 95% CI = 1.15 to 1.81, *P* < 0.00001) post intervention. However, the heterogeneity between the included studies is significant (*I*^2^=80%, *p* = 0.006). See Figure 3A for the forest plot showing the details of the result.

For FEV1/FVC, the result showed that the experimental group is not significantly better than the control (MD = 0.26, 95% CI = −0.65 to 1.16, *P* = 0.58) post intervention. In addition, there is significant heterogeneity between the included studies (*I*^2^= 86%, *p* < 0.00001). See Figure 3A for the forest plot showing the details of the result.

### Motor function

**Figure 4:**
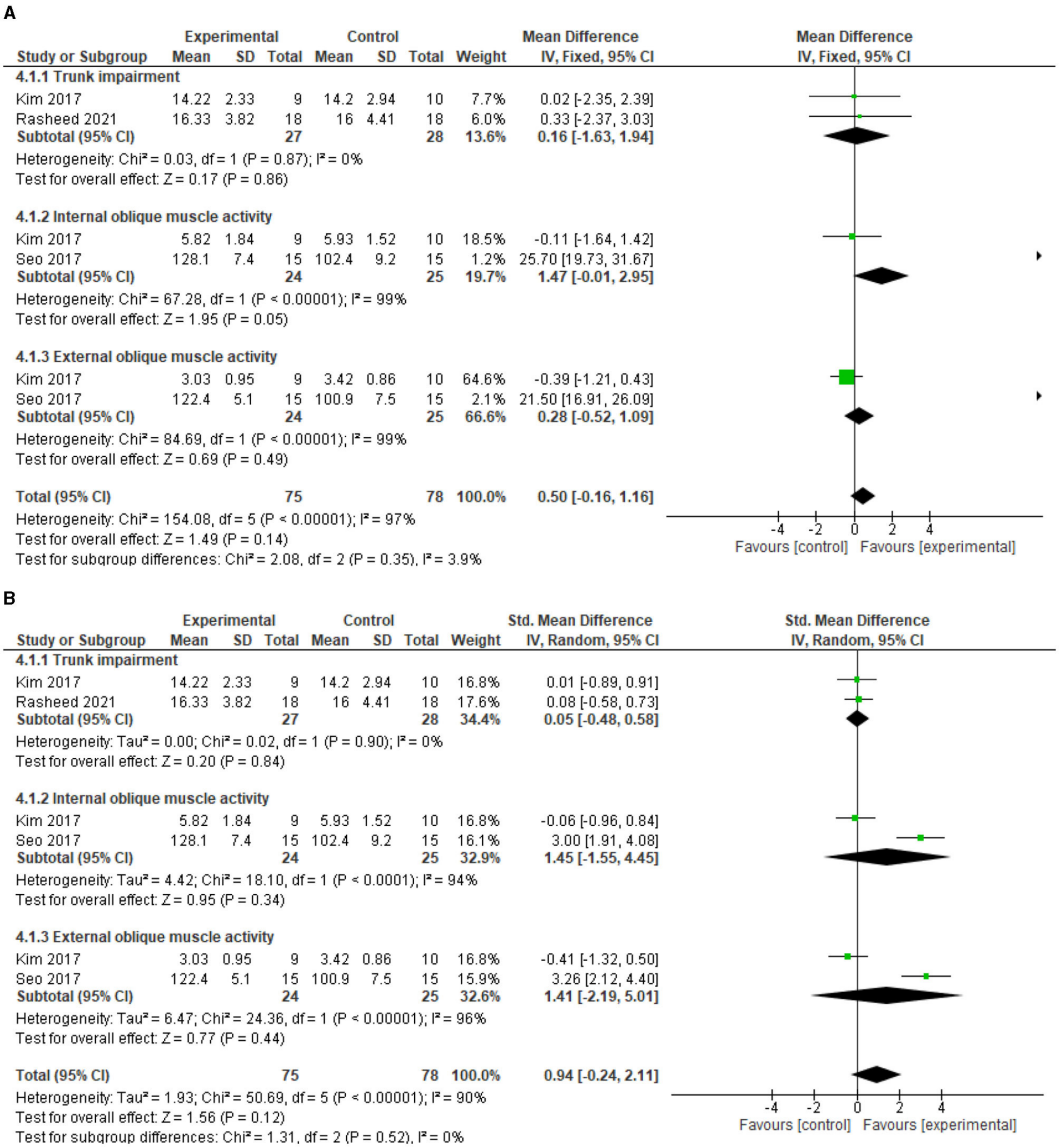
**(A)** Effect of diaphragmatic breathing exercise on motor function post-intervention. **(B)** Effect of diaphragmatic breathing exercise on motor function post-intervention (sensitivity analysis).

The test for overall effects post intervention showed that the experimental group is not superior to the control (MD = 0.5, 95% CI = −0.16 to 1.16, *P* = 0.14) post intervention, although there was a trend toward better improvement in the experimental group. In addition, there was significant heterogeneity between the included studies (*I*^2^= 97%, *p* < 0.00001). See Figure 4A for the forest plot showing the details of the result. In addition, following sensitivity analysis, the test for overall effects post intervention still showed that motor impairment of the trunk did not improve significantly higher in the experimental group compared with the control (SMD = 0.94, 95% CI = −0.24 to 2.11, *P* = 0.12). See Figure 4B for the details of the result.

For the individual motor impairment of trunk parameters, trunk impairment did not improve significantly better in the experimental group compared with the control (MD = 0.16, 95% CI = −1.63 to 1.94, *P* = 0.86) post intervention. However, the heterogeneity between the included studies is not significant (*I*^2^=0%, *p* = 0.87). See Figure 4A for the forest plot showing the details of the result.

For motor activity of internal abdominal oblique muscle, there was a trend toward better improvement in the experimental group compared to the control (MD = 1.47, 95% CI = −0.01 to 2.95, *P* = 0.05) post intervention. However, there is significant heterogeneity between the included studies (*I*^2^= 99%, *p* < 0.00001). See Figure 4A for the forest plot showing the details of the result.

For motor activity of external abdominal oblique muscle, the experimental group did not improve significantly better compared to the control (MD = 0.28, 95% CI = −0.52 to 1.09, *P* = 0.49) post intervention. In addition, the heterogeneity between the included studies is significant (*I*^2^=99%, *p* < 0.00001). See Figure 4A for the forest plot showing the details of the result.

#### Interpretation of the evidence

Based on the current available literature, there is limited evidence on the superior effect of deep breathing exercise compared with the control on FVC, FEV1, and PEF in patients with stroke. See Table 3 for the details of the interpretation of the evidence.

**Table 3:** Evidence quality assessment.

						**Number of participants**			
**Outcome**	**Number of studies**	**Risks of bias**	**Inconsistency**	**Indirectness**	**Imprecision**	**Experimental**	**Control**	**Effect size (95% CI)**	**Overall certainty of the evidence**
FVC	3	Serious	Very serious^a^	Not serious	Serious	38	38	−0.90 (0.76 to 1.04)	⊕⊕○○ Low
FEV1	3	Serious	Very serious^a^	Not serious	Serious	38	38	−0.32 (0.11 to 0.52)	⊕⊕○○ Low
PEF	3	Serious	Very serious^a^	Not serious	Serious	38	38	1.48 (1.15 to 1.81)	⊕⊕○○ Low
FEV1/FVC	2	Serious	Not serious	Not serious	Serious	28	28	0.26 (−0.65 to 1.16)	⊕⊕○○ Low
Trunk impairment	2	Serious	Not serious	Not serious	Serious	27	28	0.16 (1.63 to 1.94)	⊕⊕○○ Low

^a^significant heterogeneity.

^b^sample size < 400.

## Discussion

The aim of this study is to determine the effects of diaphragmatic breathing exercise on respiratory, cognitive, and motor functions outcomes after stroke. The results showed that diaphragmatic breathing exercise is only superior to the control at improving FVC, FEV1, and PEF. Outcomes such as the FVC, FEV1, FEV1/FVC, PEF, MIP, MEP, motor impairment of the trunk, abdominal muscles thickness, balance, and cognitive ability are required to effectively carry out the activities of daily living (ADL) (25, 48).

The ability to carry out ADL gets impaired following a stroke (49, 50). The inability to carry out ADL can result in reduced quality of life (27, 51). Thus, improving ADL and increasing quality of life are the most significant goals in stroke rehabilitation (52, 53). Diaphragmatic breathing exercise can be used to help improve respiratory function and functional outcomes that will eventually translate to improvement in the patients' ability to carry out ADL and increase in their quality of life. This is because diaphragmatic breathing helps to improve delivery of oxygen to tissues, which is important for various metabolic activities in the body (21, 54). However, it should be noted that outcomes such as trunk motor impairment, abdominal muscle thickness, balance, and cognitive capacity may also be necessary for the effective performance of activities of daily living. As a result, our findings need to be interpreted with caution.

It is also important to note that most of the studies included in the review used participants who have normal BMI and moderate disability. Mechanics of the lungs and the chest wall are altered with increasing BMI because of fat deposit in the mediastinum and the abdominal cavity (55). In addition, respiratory function has significant correlation with functional ability (56). Therefore, it is possible that most of the participants in the included studies had moderate impairment in respiratory function as well. Thus, the findings seem to suggest that diaphragmatic breathing exercise improves outcomes in patients with stroke who have moderate disability.

Although the result of the meta-analysis showed that there was no significant difference in motor function between groups post intervention, there was a trend toward better improvement in motor function in the experimental group. Thus, it is possible that the effect on motor function in favor of the experimental group was masked by confounding or control variables such as the small number of studies included in the meta-analysis and/or their small sample sizes. Small sample size can undermine the effect of an intervention (57). Similarly, for cognitive function, there was no adequate number of studies to carry out a meta-analysis. Therefore, future studies on the effect of diaphragmatic breathing exercise in patients with stroke should assess both cognitive and motor function outcomes in addition to those of respiratory function. This is because diaphragmatic breathing exercise may have the potential to enhance improvement in cognitive and motor functions after stroke.

In addition, the findings of the study should be interpreted in light of several factors. First, there is significant heterogeneity in most of the results of the study outcomes which can undermine the validity and reliability of the result (58). This heterogeneity might be caused by the use of different control group interventions, outcome measures, and probably other characteristics of the participants in the included studies. Similarly, in most of the studies, diaphragmatic breathing exercise was not practiced as a stand-alone intervention; it was combined with other forms of interventions such as the pursed-lip breathing exercise. Such exercises are reported to improve respiratory function and other outcomes after stroke (59, 60). Thus, it is probably way better if diaphragmatic breathing exercise is used as an adjunct therapy in combination with other interventions such as pursed-lip breathing and relaxation training (46).

Similarly, it is worth noting that diaphragmatic breathing exercise is not without any adverse events. This is because adverse events such as dizziness and fatigue can occur during breathing exercises (41). Therefore, it should be administered with caution, close monitoring, and supervision. In addition, in most of the studies, the intensity of diaphragmatic breathing exercise used is not clear. Lack of clarity in the protocol of an intervention can affect its reproducibility; and clinical replicability must be considered in RCTs (61). Therefore, it is important that studies determining the effects of diaphragmatic breathing exercise on outcomes after stroke be standardized and well-controlled.

Furthermore, although the present study has some strengths such as extensive literature search of multiple databases, it is also not without limitations. One of these limitations is the exclusion of any literature published in languages other than English. This exclusion might have limited the contributions of many quality studies on the subject matter. Additionally, we included studies with variability in the protocols of breathing exercise, wherein some studies used breathing exercise in addition to other intervention which is another potential limitation that can affect the generalizability of this study. Thus, interpretation of the findings of this study should be made in light of all its potential limitations.

## Conclusion

There is limited evidence that diaphragmatic breathing exercise may help improve FVC, FEV1, and PEF which may in turn enhance recovery of function post stroke. Therefore, there should be more consideration given to implementing of diaphragmatic breathing exercise for the rehabilitation of some patients with stroke. However, the significant statistical heterogeneity between studies in some of the outcomes should be noted when interpreting the findings of this study. Thus, standardized and well-controlled RCTs should be conducted to further determine this effect, required intensity, and the most suitable protocol.

## Data availability statement

The original contributions presented in the study are included in the article/Supplementary material, further inquiries can be directed to the corresponding author.

## Author contributions

Design, data collection, and critical review of the manuscript: AA, TW, and SN. Writing of the draft: AA. All authors contributed to the article and approved the submitted version.
