# Supplementary material for: Efficacy of diaphragmatic breathing exercise on respiratory, cognitive, and motor function outcomes in patients with stroke: a systematic review and meta-analysis
Source: Front Neurol. 2024 Jan 12;14:1233408. doi: 10.3389/fneur.2023.1233408 (PMC10811179; doi:10.3389/fneur.2023.1233408)
Supplement: Supplementary file 1 [file Data_Sheet_1.pdf]

Appendix: Search strategy

PubMED

(((((Stroke) OR (ischaemic stroke)) OR (haemorrhagic stroke)) OR (brain infarction) AND ((randomized controlled trial[Filter]) AND (humans[Filter]) AND (english[Filter]) AND (all adult[Filter]))) AND ((((Breathing exercises) OR (diaphragmatic breathing exercise) or (Respiratory muscle training)) OR (Respiratory therapy)) AND ((randomized controlled trial[Filter]) AND (humans[Filter]) AND (english[Filter]) AND (all adult[Filter])))) AND (((((((((Forced vital capacity) OR (Timed vital capacity)) AND (maximal expiratory pressure)) OR (maximal respiratory pressure)) OR (maximal inspiratory pressure)) AND (Cognition)) OR (cognitive function)) AND (movement)) OR (motor activity) AND ((randomized controlled trial[Filter]) AND (humans[Filter]) AND (english[Filter]) AND (all adult[Filter]))) AND ((randomized controlled trial[Filter]) AND (humans[Filter]) AND (english[Filter]) AND (all adult[Filter]))

EMBASE

(((((('stroke'/exp OR stroke OR (ischaemic AND stroke) OR (haemorrhagic AND stroke) OR (brain AND infarction)) AND breathing AND exercises OR (respiratory AND muscle AND training) OR (respiratory AND therapy)) AND forced AND vital AND capacity OR (timed AND vital AND capacity)) AND maximal AND expiratory AND pressure OR (maximal AND respiratory AND pressure) OR (maximal AND inspiratory AND pressure)) AND cognition OR (cognitive AND function)) AND movement OR (motor AND activity)) AND [randomized controlled trial]/lim

Scopus

(ALL (stroke) OR ALL (ischaemic AND stroke) OR ALL (haemorrhagic AND stroke) OR ALL (brain AND infarction) AND ALL (breathing AND exercises) OR ALL (respiratory AND muscle AND training) OR ALL (respiratory AND therapy) AND ALL (forced AND vital AND capacity) OR ALL (timed AND vital AND capacity) AND ALL (maximal AND expiratory AND pressure) OR ALL (maximal AND respiratory AND pressure) OR ALL (maximal AND inspiratory AND pressure) AND ALL (cognition) OR ALL (cognitive AND function) AND ALL (movement) OR ALL (motor AND activity)) AND (LIMIT-TO (EXACT KEYWORD , "Human") OR LIMIT-TO (EXACT KEYWORD , "Humans") OR LIMIT-TO (EXACT KEYWORD , "Article") OR LIMIT-TO (EXACT KEYWORD , "Controlled Study") OR LIMIT-TO (EXACT KEYWORD , "Adult"))

WoS

Stroke (All Fields) OR ischaemic stroke (All Fields) OR haemorrhagic stroke (All Fields) OR brain infarction (All Fields) AND Breathing exercises (All Fields) OR Respiratory muscle training (All Fields) OR Respiratory therapy (All Fields) AND Forced vital capacity (All Fields)

OR (All Fields) Timed vital capacity (All Fields) AND maximal expiratory pressure (All Fields) OR maximal respiratory pressure (All Fields) OR maximal inspiratory pressure (All Fields) AND cognition (All Fields) OR cognitive function (All Fields) AND movement (All Fields) OR motor activity (All Fields)

CENTRAL (only CINAHL sources)
Stroke in All Text AND Breathing exercises in Title Abstract Keyword OR Diaphragmatic breathing exercise in Title Abstract Keyword OR Respiratory muscle training in Title Abstract Keyword OR Respiratory therapy in Title Abstract Keyword - in Trials (Word variations have been searched)

PEDro
Stroke and Breathing exercse
